# Association between Air Pollution and Adverse Pregnancy Outcomes in Vancouver

**DOI:** 10.1289/ehp.112-a792a

**Published:** 2004-10

**Authors:** John A. Bukowski

**Affiliations:** ExxonMobil Biomedical Sciences, Inc., Annandale, New Jersey, E-mail: john.a.bukowski@exxonmobil.com

In the November 2003 issue of *EHP*, Liu et al. (2003) concluded that “relatively low concentrations of gaseous air pollutants are associated with adverse effects on birth outcomes.” Although this may be true from a purely statistical sense, there appear to be limitations of this research that suggest cautious interpretation of the findings.

Liu et al. (2003) evaluated individual-level birth certificate data, which is an improvement over the ecologic designs of past time-series studies on pollution. However, birth records do not contain most of the variables that are important predictors of low weight and preterm births. These include smoking, alcohol and/or drug abuse, low socioeconomic status (SES), small maternal weight or height, complications of the current or previous pregnancy [e.g., pregnancy-induced hypertension, previous low birth weight (LBW), spontaneous abortion], insufficient weight gain during pregnancy, maternal illness (e.g., fever), and job-related exertion (Berkowitz and Papiernik 1993; Holmes and Soothill 1996; Kramer 1987, 2003; Lang et al. 1996; Moore 2003). Many of these are major factors that substantially affect risk. For example, maternal smoking during pregnancy, which has a prevalence of 10–20% in the United States (Ebrahim et al. 2000; O’Campo et al. 1995), is associated with a 2- to 4-fold increase in risk of LBW or growth restriction (Kramer 1987; Lang et al. 1996; Nordentoft et al. 1996). Therefore, there is considerable room for uncontrolled confounding that might account for the small odds ratio of 1.05–1.10 observed by Liu et al. (2003).

Liu et al. (2003) argued that uncontrolled or residual confounding is an unlikely explanation for their results because *a*) there is no evidence that these factors are associated with air pollution; *b*) ecologic measures of SES did not modify the associations; and *c*) “there were only slight differences between crude and adjusted estimates,” and “individual characteristics ... did not attenuate the risk estimates.” However, these arguments have limitations.

First, there may not be evidence that important risk factors co-vary with pollution, but it seems reasonable that many might correlate with residential location. Liu et al. (2003) linked pollution measurements in 13 census subdivisions to births within those subdivisions. If gaseous pollutant measurements and other factors (e.g., SES, smoking prevalence) co-vary by census subdivision, then confounding could occur. Second, ecologic measures are poor surrogates for individual-level ones, which can result in confounder misspecification and residual confounding (Greenland 1980; Liu 1988; Marshall and Hastrup 1996; Morgenstern 1998). Third, the individual-level covariates included in some of the models did appear to have substantive impacts. For example, the odds ratio for the association between LBW and first-month sulfur dioxide exposure changed from a crude value of 0.95 to a significant 1.11 after adjustment for confounding. This is a 17% absolute increase in risk and a change in coefficient from –0.05 to +0.10 per 5 ppb. In other instances the adjustment caused a significant elevation to become a deficit (e.g., association between preterm birth and first-month exposure to ozone) or a null value to become a significant protective effect (preterm birth and last-month ozone exposure). This apparent impact of confounding was caused by variables (e.g., maternal age and season of birth) that are weaker risk factors than many missing variables, such as smoking, SES, and weight gain (Berkowitz and Papiernik 1993; Kramer 1987; Lang et al. 1996). This suggests considerable potential for residual confounding.

The findings of Liu et al. (2003) also lack biological coherence with the literature. The authors invoked a biological mechanism for air pollution similar to cigarette smoking. For smoking, the risk is predominantly during the third trimester, primarily from decreased fetal growth, which has been attributed to decreased maternal and fetal nutrition among smokers and hypoxia from inhaled carbon monoxide (Holmes and Soothill 1996; Kramer 1987; Petridou et al. 1990). However, most of the significant increases reported by Liu et al. (2003) were associated with exposures during the first month or trimester, with no effects seen during the third trimester. It is unclear how these early, low-level pollution exposures, which lack the substantive impact of smoking, would alter fetal growth.

Liu et al. (2003) also do not discuss the potential for spurious results due to multiple comparisons. The authors reported 36 associations within the tables, and many more were likely performed, including multipollutant models. Therefore, at least some of the significant results may be due to chance.

In conclusion, the above limitations could easily account for the findings reported by Liu et al. (2003), without invoking novel effects from air pollution.
